# Redox Sensitive Cysteine Residues as Crucial Regulators of Wild-Type and Mutant p53 Isoforms

**DOI:** 10.3390/cells10113149

**Published:** 2021-11-12

**Authors:** Elena Butturini, Giovanna Butera, Raffaella Pacchiana, Alessandra Carcereri de Prati, Sofia Mariotto, Massimo Donadelli

**Affiliations:** Department of Neurosciences, Biomedicine and Movement Sciences, Section of Biochemistry, University of Verona, 37134 Verona, Italy; elena.butturini@univr.it (E.B.); giovanna.butera@univr.it (G.B.); raffaella.pacchiana@univr.it (R.P.); alessandra.carcererideprati@univr.it (A.C.d.P.)

**Keywords:** cancer, p53, mutant p53, redox, oxidative stress, post-translational modifications

## Abstract

The wild-type protein p53 plays a key role in preventing the formation of neoplasms by controlling cell growth. However, in more than a half of all cancers, the *TP53* gene has missense mutations that appear during tumorigenesis. In most cases, the mutated gene encodes a full-length protein with the substitution of a single amino acid, resulting in structural and functional changes and acquiring an oncogenic role. This dual role of the wild-type protein and the mutated isoforms is also evident in the regulation of the redox state of the cell, with antioxidant and prooxidant functions, respectively. In this review, we introduce a new concept of the p53 protein by discussing its sensitivity to the cellular redox state. In particular, we focus on the discussion of structural and functional changes following post-translational modifications of redox-sensitive cysteine residues, which are also responsible for interacting with zinc ions for proper structural folding. We will also discuss therapeutic opportunities using small molecules targeting cysteines capable of modifying the structure and function of the p53 mutant isoforms in view of possible anticancer therapies for patients possessing the mutation in the *TP53* gene.

## 1. Introduction

Oxidative damage to proteins has a critical role in promoting several disorders such as degenerative diseases and cancer [1,2]. Under oxidative stress conditions, several key redox sensitive amino acids can be modified by reactive oxygen and nitrogen species (ROS and RNS), affecting the structure, activity or ligand binding capacity of targeted proteins [3]. Cysteine residues of proteins have both structural and regulatory roles and are particularly susceptible to oxidation [4]. This reactivity leads the cysteine residues to act as redox-sensitive molecular sensors or switches with catalytic activity and metal binding capacity that are oxidative status-dependent [4,5]. Thus, cysteine redox modifications have an important role in allowing proteins to respond to ROS, controlling redox homeostasis and the ROS-mediated cellular pathway [5].

Several proteins like the transcription factor p53 are regulated via redox mechanisms [6]. The *TP53* tumor suppressor gene encodes a DNA-binding protein that regulates numerous cellular processes like cell growth and cell death [7]. The p53 DNA-binding ability is dependent on wild-type conformation of the protein and is regulated by post-translational modifications [8]. A wide variety of human cancers carry *TP53* mutations, most commonly missense mutations [9]. Mutant p53 proteins generally lose DNA binding capability of the tumor suppressor wild-type p53, acquiring additional oncogenic activities called gain-of-function activities (GOFs) [10]. Several studies reported that, in contrast with the wild-type protein, mutant p53 isoforms counteract antioxidant activity and enhance intracellular ROS, influencing the cellular redox balance and promoting cancer survival [11]. The DNA-binding core domain of wild-type p53 has key cysteine residues, crucial for the protein structure and function. From a therapeutic perspective, p53 cysteine residues are important targets of novel compounds that refold missense mutant p53 binding to specific p53 cysteine residues [6]. In this review, we summarize the redox-sensitive cysteine residues of wild-type and mutant p53, describing cysteine oxidative post-translational modifications to highlight their importance as target therapy for promoting p53 correct folding and activity and regulating mutant p53 oxidative responses.

## 2. Structure and Function of Wild-Type p53 and of Its Mutant Counterpart

**Figure 1:**
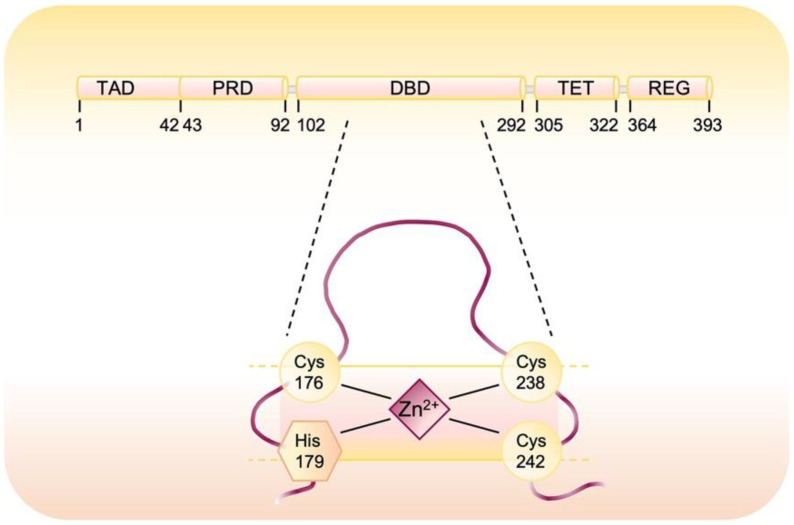
Domain structure of p53. Human p53 is composed of 393 amino acid residues and has an N-terminal transactivation domain (TAD), proline-rich domain (PRD), DNA-binding domain (DBD), tetramerization domain (TET), C-terminal regulatory domain (REG). The magnification shows the residues involved in the coordination of a zinc ion.

The oncosuppressor p53 protein acts as a transcription factor and contains different functional domains: (i) N-terminal transactivation domain (TAD) which interacts with the transcriptional machinery; (ii) proline rich-region (PRD), which is required for p53 stabilization; (iii) DNA binding domain (DBD), in which the responsive element binds proteins such as MDM2 and 53BP1 that, respectively, positively or negatively affect p53 activity; (iv) oligomerization domain (TET) which is essential for tetramer formation and represents the active form of p53 and (v) C-terminal regulatory domain (REG), containing post-translationally modified residues involved in modulation of protein stability [12]. Specifically, the DNA-binding core domain of the p53 protein has 10 cysteine (Cys) residues [13], three of them, Cys 176, Cys 238, and Cys 242, together with His179, hold a divalent zinc atom that is crucial for the correct folding of p53 and the stabilization of loop/helical structure of the core domain in the wild-type conformation (Figure 1) [13,14]. The p53 tetramer coordinates a single Zn^2+^ in each one of its four equal subunits. Zinc chelation disrupts this architecture due to oxidation of several cysteines and formation of disulfide-bound protein aggregates [15]. The intracellular concentration of free zinc ions modulates p53 activity and stability but the excess or loss of zinc alter the p53 protein structure and compromise DNA binding and transcriptional activity [15,16]. Upon activation, p53 directly regulates the transcription of around 500 genes and indirectly regulates several additional genes and thereby controls diverse cellular processes [17]. On the basis of the cell type and the type of cellular stress, downstream targets are transcribed. p53, as a tumor suppressor, transcriptionally regulates a lot of target genes that are implicated in various biological processes including DNA damage repair (i.e., GADD45α, PCNA), cell cycle arrest (i.e., CDKN1A), senescence, and apoptosis (i.e., Puma, Noxa, Bax, Bid, Casp1, 6 and 10) [18]. p53 is also able to influence cell metabolism decreasing both glycolytic rate (i.e., TIGAR, GLUT1) and protein synthesis (i.e., SESN1, SESN2, SESN3) lowering ROS at the same time [19,20]. Moreover, a number of transcriptional targets of p53 are directly or indirectly involved with redox homeostasis. Among the redox controlling genes, specifically upregulated are glutathione peroxidase (GPX), p53 induced genes (PIGs), and glutaredoxin 3 (Grx3) [21].

Another important aspect is that oxidation of p53 or chelation of Zn^2+^ turned wild-type p53 into a form functionally similar to mutant p53 as described in the His 175 mutant where the mutation disrupts normal zinc/redox dependent folding [22,23].

Under the non-stressed condition, wild-type p53 protein is maintained at a low level in cells by the proteasome degradation pathway [17]. In response to a wide variety of stress signals, the p53 protein is stabilized through post-translational modifications and it becomes able to promote the coordinated expression of many target genes through the binding to specific DNA sequence in the regulatory regions of its target genes [24]. In this way, p53 regulates a wide range of cellular biological processes to maintain genomic integrity and prevent tumor formation, including antioxidant defenses as described in the next paragraph.

### 2.1. Wild-Type p53 Regulates Redox Balance

Several types of stress cause ROS accumulation and p53 protects the oxidation of the genome by ROS that are the major cause of genetic instability [25,26]. The regulation of oxidative stress by p53 is complex, exerting both pro-oxidative and anti-oxidative effects [27]. The result of p53 activation is dependent on severity and duration of the stress [26]. Under severe stress conditions, p53 induces cell death or cell cycle arrest [28]. There are several p53-inducible proteins that are activated during apoptotic responses and promote ROS generation such as p53-induced gene 3 (PIG3) [28], BAX, PUMA to support pro-apoptotic activity of p53 in response to severe stress [29]. Furthermore, to enhance this effect, p53 can also modulate the expression of genes such as superoxide dismutase 2 (SOD2) [30] and glutathione peroxidase 1 (GPX1) [31] inhibiting them from increasing oxidative stress and support the pro-apoptotic activity of p53 [28]. On the contrary, under low-stress conditions p53 does not induce cell death and suppresses ROS production [32]. Regarding antioxidant roles, wild-type p53 regulates several signaling pathways to exert antioxidant activities [24]. For instance, some studies reported that wild-type p53 suppresses the expression of NOX4, a catalytic subunit of the NADPH oxidase complex that catalyzes the molecular oxygen reduction to different types of ROS, inhibiting ROS production [33]. Another important p53-activated antioxidant genes include the enzymes specifically involved in ROS degradation such as GPX1 [34] or catalase [35]. Sestrins are p53-target genes involved in antioxidant response and their inactivation by p53 mutation or other mechanisms has a critical role in carcinogenesis [36]. Furthermore, p53 can exert its anti-oxidant roles preventing the inflammatory events through the inhibition of the NF-κB activity, that is a transcription factor and crucial regulator of the expression of chemokines [37]. P53 can also inhibit ROS production by metabolic changes as the activation of p53-induced glycolysis and apoptotic regulator TIGAR that inhibit glycolysis and decrease ROS levels [19]. The importance of p53 induced antioxidant function is supported in p53-deficient cells that result in excessive oxidation of DNA and increased mutation rate [32]. Intriguingly, these wild-type p53-induced responses are in line with its tumor suppressor role, while its p53 mutant counterpart generally exerts an opposite response to sustain its oncogenic role in cancers, such as the pro-oxidant effect or reprogramming of energy metabolism and chemoresistance, which are briefly discussed in the following sections.

### 2.2. Mutant p53 Gain-of-Function Structure and Roles in Cancer

Mutations in the *TP53* gene are among the most common gene-specific alterations in human cancers [9]. The frequency of *TP53* gene mutations can vary widely among cancer types, reaching over 70% in ovarian and pancreatic cancers [38]. The majority of p53 mutations in human cancers are missense mutations, which usually result in the expression of full-length mutant p53 proteins [39]. The majority of mutations occurs in the p53 DNA-binding domain, resulting in the loss of DNA-binding activity of mutant p53 [39]. In addition to a loss of canonical p53 role, the most common mutants acquire new different functions (Gain-Of-Functions, GOF) that fuel tumor progression [39,40]. Most p53 missense mutations occur at six ‘mutational hot-spots’ in the DNA-binding domain of p53, including R175, G245, R248, R249, R273 and R282 residues, and correlate with poor cancer-free survival [41,42]. Two main types of mutant “hotspot” sites are named contact mutants, that include mutations in residues directly involved in DNA binding, and conformational mutants, such as mutations that cause local or global conformational distortions [40]. As part of its GOF, mutant p53 interacts with different proteins to enhance or inhibit their activities [43]. While wild-type p53 protein is kept at a low level in cells by the proteasome degradation pathway under non-stressed conditions, mutant p53 protein usually accumulates to a high level in tumors [41].

A number of studies report that GOF p53 promotes tumor progression by regulating several diversified pathways involved in reprogramming of metabolism in responses to cancer-related stressing conditions, in sustaining oncogenic oxidant intracellular environment and promoting chemotherapy [11,44,45]. Indeed, in contrast to the antioxidant role of wild-type p53, mutant p53 proteins can sustain ROS production through several mechanisms that are further described in the following paragraph.

### 2.3. Mutant p53-Induced Oncogenic Mechanisms to Promote ROS Production

**Figure 2:**
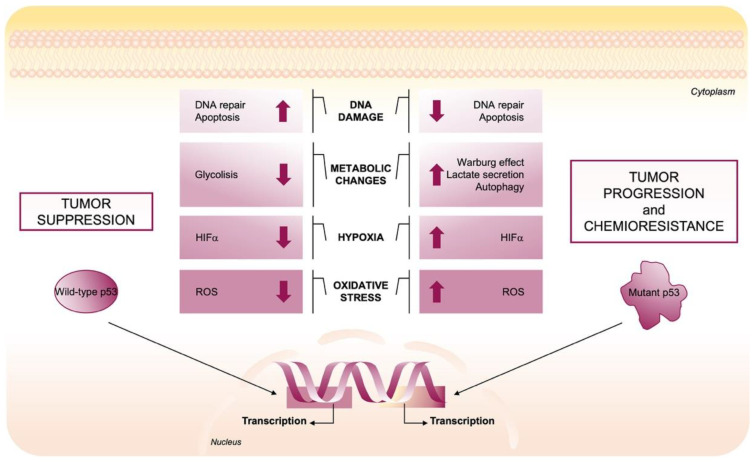
Role of wild-type and mutant p53 in the tumor microenvironment. The p53 tumor suppressor maintains a long-term anti-cancer environment in normal cells by tuning cell metabolism and ROS levels, maintaining the genomic stability and microenvironment. Wild-type p53, via its target genes, regulates cellular metabolism mainly by enhancing DNA repair, suppressing glycolysis, decreasing HIFα expression, and modulating ROS levels. In contrast, mutant p53 enhances tumor initiation, progression, and invasiveness by suppressing DNA repair, inhibiting apoptosis, inducing Warburg Effect and ROS production.

Several studies summarized that wild-type p53 and its mutant counterpart regulate oxidative stress in opposite ways [11]. As described in the previous section, under low stressing conditions, wild-type p53 prevents ROS production by inducing the expression of many antioxidant enzymes [32]. On the contrary, mutant p53 sustains an increase in intracellular ROS by alteration of several molecular pathways to favor genomic instability and sustain tumor progression [11]. Regarding the metabolic changes, mutant p53, in contrast to its wild-type form, stimulates aerobic glycolysis for energy production, a phenomenon known as the Warburg effect, through several responses: (i) promoting the translocation of GLUT1 (glucose transporter 1) to plasma membrane [46]; (ii) inhibiting AMPK that negatively regulates the Warburg effect by the repression of the hypoxia-induced factor 1 (HIF1) pathway [47,48]; (iii) maintaining the glycolytic enzyme GAPDH in the cytosol which has a critical impact on the anti-apoptotic and anti-autophagic effects driven by mutant p53 and stimulates glycolysis, lactate secretion and chemoresistance (Figure 2) [49]. Furthermore, mutant p53 proteins repress the transcription of sestrines’ antioxidant protein family, and consequently AMPK/PGC-1α/UCP2 blockage stimulating mitochondrial O_2_^−^ production and contributing to the pro-oxidant and oncogenic effects of mutant p53 [36]. The importance of SESNs and AMPK proteins in the maintenance of metabolic and redox homeostasis in cells is revealed by their alteration that leads to increased oxidative stress and tumor progression [36,49,50]. In addition, through alteration of the SESN/AMPK axis, mutp53 promotes autophagy defects in cancer cells [45]. Furthermore, the overexpression of various tumor-associated p53 mutants can render cancer cells more resistant to the effect of chemotherapeutic drugs [41,42], whereas knockdown of endogenous mutant p53 sensitizes cancer cells to killing by such molecules [51]. Interestingly, Torrens-Mas et al. revealed that mutant p53-induced oxidative stress is tightly regulated to keep the ROS increase moderate to promote cancer cell survival [52].

The interference with pivotal signaling pathways are important mechanisms through which p53 mutants exert their oncogenic functions and each of these diversified pathways regulated by mutant p53 might provide new therapeutic opportunities in order to counteract chemoresistance in cancer patients bearing mutant *TP53* gene.

## 3. Oxidative Post-Translational Modifications of Proteins Cysteines

Evidence in the literature describes that ROS/RNS may function as signaling molecules in many cellular processes through the covalent modification of redox-sensitive proteins [53,54]. Depending on types, amounts and cellular localization of oxidants, proteins undergo different reversible or irreversible oxidative post-translational modifications that affect protein secondary and tertiary structure and ultimately their functions [55,56].

Due to the redox chemical properties related to the thiol group, Cys is one of the most redox sensitive residues in proteins and the oxidative post-translational modifications of Cys have emerged as regulatory elements of the physiology and pathophysiology of cells. Cys is an amino acid with a unique chemistry and the knowledge of sulfur chemistry account for the observation that Cys residues are preferentially oxidized in cells. First, a sulfur atom may exist in different redox states, and this is certainly useful in redox biology regulation. Then, the thiol group can be deprotonated to a thiolate anion acquiring more nucleophilic characters and higher redox sensitivity. The ionization acid constant (pKa) of SH group determines the equilibrium between thiol and thiolate forms and its value is influenced by protein microenvironment. Usually, the SH group in most of proteins Cys residues is higher than eight so that, in the reducing environment of cells, it remains almost completely protonated and is not able to react with ROS/RNS. However, the neighboring positively charged amino acid residues (i.e., Arginine, Histidine, and Lysine) may perturb the pKa values of Cys and influence their reactivity. In this environment, reactive cysteines have a lower pKa and exist predominantly in the thiolate form which is more subjected to oxidation [56,57,58].

**Figure 3:**
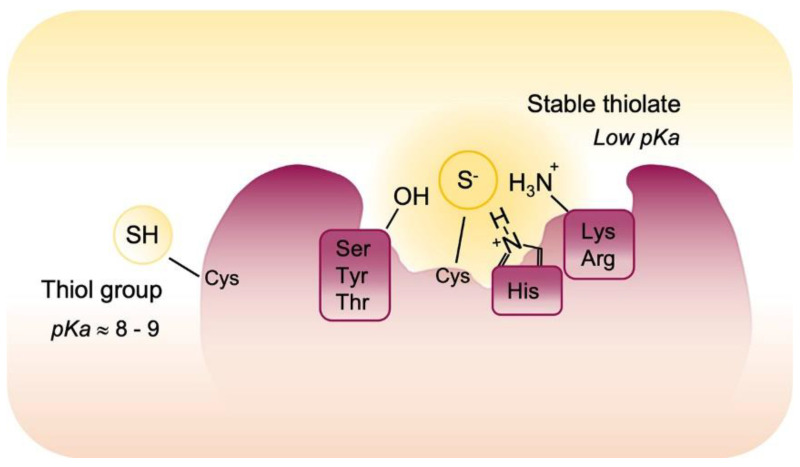
Reactivity of cysteines. The pKa value of most of proteins Cys residues have a pKa value higher than 8 but some redox sensitive cysteine residues are localized in specific protein environment that contributes to decrease their pKa. Specifically, neighboring amino acids stabilized protein thiolates via hydrogen bonds and electrostatic effects and render them sensitive to oxidative post-translational modifications.

Moreover, redox sensitive Cys residues are closest to the amino acid as serine, threonine and tyrosine, which promote H bond formation and stabilize the thiolate anion. Finally, the thiols of redox active Cys are exposed within the three-dimensional structure of the protein (Figure 3). These Cys residues or redox active cysteines are “redox sensors” that turn between thiols and thiolates in response to modified redox microenvironment and react with ROS/RNS to form reversible or irreversible post-translational modifications and this may determine the fate of the protein and in some cases of the whole cell.

Under oxidative stress, the thiolate anion of redox sensitive Cys may be reversibly oxidized to sulfenic acid (PS-OH) or may react with vicinal thiol to form intra- or intermolecular disulfide (PS-SP or PS-SP’) or mixed disulfide when react with low mass thiol (PS-SX). Importantly, when this low mass thiol is glutathione (GSH), protein is glutathionylated (PS-SG) [59,60]. The sulfenic acid is very unstable and can represent the first step to protein disulfide bond formation or, under strong oxidant stress can be irreversible oxidized. Reactive nitrogen species such as NO may nitrosylate Cys and form nitrosyl Cys that is unstable and in the presence of GSH may evolve into the S-glutathionylated protein. These disulphide bonds can alter protein structure and function, but they can be reduced back to free thiol by the thiol disulfide exchange reaction catalyzed by oxidoreductase, such as thioredoxin or glutaredoxin.

**Figure 4:**
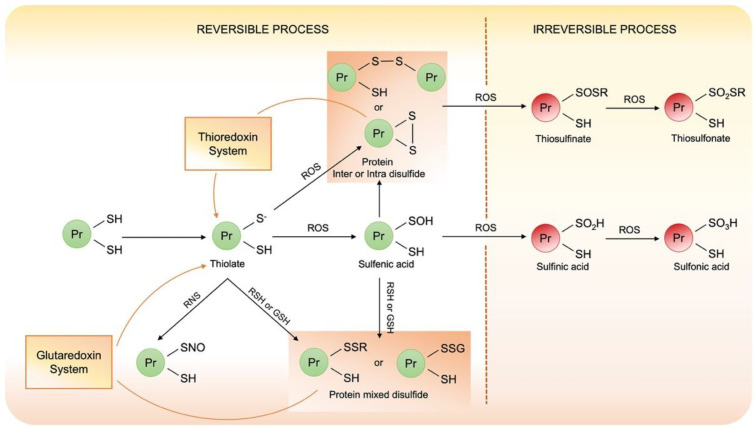
Schematic representation of cysteines reactivity under oxidative stress.

Under strong oxidative conditions, protein thiols evolve in the formation of irreversible post-translational modifications. These PTMs are associated with protein misfolding and aggregation. These covalent aggregates are not reduced back even when the redox conditions are restored so that these irreversible PTMs are associated with oxidative damages. A summary of the various oxidative post-translational modifications of Cys associated with their formation is presented in Figure 4.

### Oxidative Post-Translational Modifications of p53

p53 is regulated by several PTMs both during normal homeostasis and in stress-induced responses. p53 lies at the center of a network of complex redox interactions and the direct redox regulation of the protein is emerging as a means to control the induction of transcriptionally active wild-type p53 by a variety of stress-related signals (DNA damaging agents, hypoxia, heat shock, etc.). Human p53 contains 10 Cys residues located in the central DNA-binding core domain, nine of which are highly conserved (all but Cys 229) [61] and three of which are involved in the coordination of zinc ions (Cys 176, 238, and 242, along with His 179) [62] and are important to maintain the p53 structure.

Zinc binding has been shown to be crucial for p53 activity and its removal by chelating reagents or oxidative stress results in rapid unfolding of the protein and loss of DNA-binding activity [22,63]. Recent studies demonstrated that a strong reducing microenvironment is required for p53 binding to DNA in vitro and single point-mutation of any of the three zinc-binding Cys or their modification induced by oxidative stress results in conformational changes and loss of p53 DNA-binding abilities [64]. However, the specific Cys residues modified during p53 oxidation and the redox-specific mechanisms involved are poorly understood. Cho et al. [62] analyzed the crystal structure of residues 94-289 of p53 bound to DNA in order to identify which of the 10 Cys residues in this domain may be exposed to the solvent and more easily redox regulated, revealing that Cys 124, Cys 176, Cys 182, Cys 229, Cys 242 and Cys 277 can theoretically react with small molecules on the surface of p53 and are likely more prone to oxidation hampering DNA binding. Moreover, either Cys 176 and Cys 242 can virtually form a disulfide bond with Cys 238 after zinc removal (Figure 1). Scotcher et al. used mass spectrometry and top-down fragmentation to study the oxidation pathways in the p53 core domain demonstrating that the zinc coordination site is the initial target for ROS-induced oxidation and that an intramolecular disulfide bond between Cys 182 and any of the three zinc-coordinating Cys (Cys 176, 238 and 242) is accompanied by the release of zinc and the breakdown of the regular structure of the protein [65]. Cys 182 was confirmed by Held et al. to be the most susceptible residue to diamide oxidation also within the cells [66].

Other evidence that p53 is also sensitive to redox regulation in vivo has been provided since the hydroxyl radical produced by copper is able to oxidize p53 Cys thiol groups [67] and cells exposed to hydrogen peroxide results in decreased transactivation by p53 of a target reporter gene construct in vivo [68]. Interestingly, the result of p53 regulation by direct alteration of p53 Cys residue oxidation is complex and multifaceted as changes in Cys 277 redox state results in a differential regulation of GADD45 allowing p53 to discriminate among individual response elements (REs), according to their sequence, representing another mechanism to control p53 sequence specific DNA binding [69].

**Figure 5:**
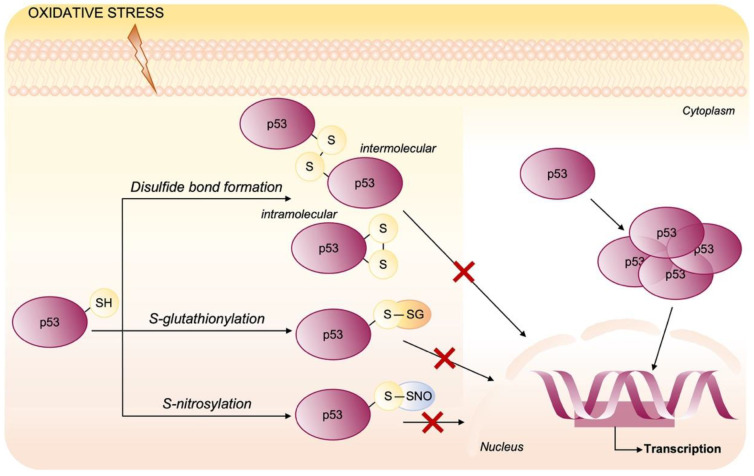
Oxidative post-translational modifications of p53. In p53, the reduced cysteine residues located in the DNA-binding domain are important to maintain the p53 tetrameric structure. p53 activity is known to be affected by several post-translational modifications induced by oxidative stress that impair the DNA binding. Cysteine residues can be easily oxidized, and their oxidation is mostly reversible resulting in intermolecular/intramolecular disulfide bond formation, S-glutathionylation and nitrosylation.

Several lines of evidence show that p53 is subjected to glutathionylation in vitro and mass spectrometry of GSH-modified p53 protein identified the Cys 124, 141 and 182 as the sites of glutathionylation, with Cys 141 being probably the most reactive (Figure 1). In human tumor cells (HCT116 colon cancer cells) glutathionylated p53 protein was detected among the proteins precipitated by anti-GSH antibodies and the modified p53 has significantly reduced ability to bind its consensus DNA sequence [70]. Additionally, Cys 277, highly reactive to N-ethylmaleimide (NEM) and surface exposed, was demonstrated to be likely a site of oxidative glutathionylation contributing to the negative regulation of p53 [71]. Inactivation of p53 DNA binding was associated also with modification by RNS that can result in S-nitrosylation of Cys [72] or tyrosine nitration (Figure 5) [73]. In the human glioblastoma multiform, concentrations of peroxynitrite consistent with those found in a hypoxic inflammatory microenvironment, are able to inactivate p53-specific DNA binding of cells in culture due to tyrosine nitration of wild-type p53 protein [74]. Otherwise, nitration of Tyr 327 stimulates the oligomerization and nuclear retention of p53 [75].

To the best of our knowledge, the function of most PTMs for mutant p53 proteins is less clear and likely their roles are similar to PTMs in wild-type p53, but this remains to be determined. Recent studies indicate contributions for phosphorylation of Ser 6, Ser 9, and Thr 81 toward a GOF for at least some mutants. For most other modifications, the current literature still cannot precisely understand their effects.

## 4. Targeting Cysteines as a Strategy to Reactivate Mutant p53

Therapeutic targeting of p53 in cancer is a promising strategy with a significant implication on cancer therapy in the future. There are three kinds of strategies aiming to develop drugs to hit mutants p53 expressed at high levels in tumor cells: to reactivate the wild-type function by promoting proper folding and stabilization of mutants, to promote its degradation, and immunotherapies based on mutant p53 neoantigen recognition.

**Figure 6:**
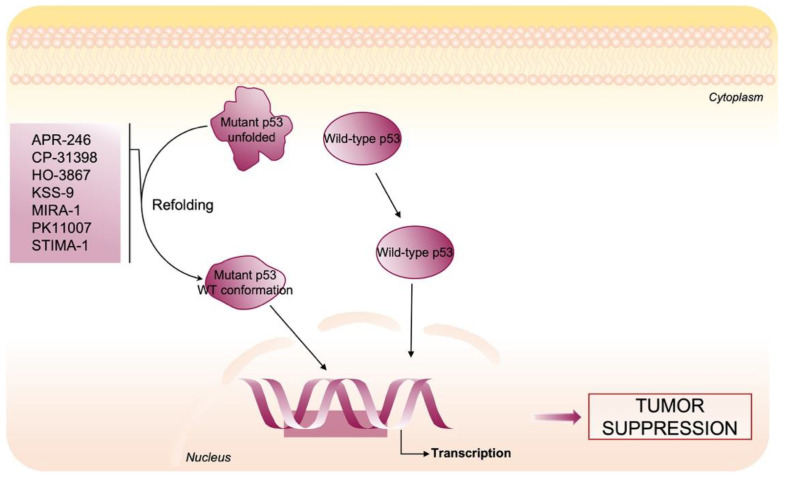
Therapeutic strategy to restore wild-type activity to mutant p53. Several small molecules have been developed to restore wild-type conformation and function to mutant p53 proteins through covalently binding to cysteines.

In this section, we focus on compounds that target the strongest nucleophiles Cys in mutant p53 in order to stabilize p53 native conformation and restore DNA binding, rescuing “wild-type like transcriptional functions” and leading to cell death and tumor suppression (Figure 6).

The core domain of p53 contains 10 Cys that are not equally reactive. As described above, thiol reactivity is affected by the local microenvironment, accessibility to solvent and steric factors [76]. Cys 277 and Cys 182 are placed on the protein surface and are suitable for electrophilic attack, while Cys 135, Cys 141, Cys 176 and Cys 275 are in an area with less availability to solvent [77]. Moreover, Cys 176, Cys 238, and Cys 242 play a major role in maintaining the correct protein folding since they coordinate the binding of a zinc atom [62,78,79]. Interesting, mutations that perturb the folding and structure of p53 can expose Cys residues that are normally buried in the wild-type. Since it is known that the redox status of Cys in p53 is relevant for its function, p53 mutants are more susceptible to oxidation that results in the formation of inter- and intramolecular disulfide bridges producing large inactive aggregates with the loss of active conformation [22,80].Hence, the idea that alkylation of thiol groups may play a role in mutant p53 rescue. In this regard, a large group of molecules able to bind the reactive Cys have been drawn: these compounds are soft electrophiles with thiol binding properties [81] due to their ability to participate in the reaction of nucleophilic addition called the Michael addition [82]. Several mutant p53-reactivating compounds are listed in Table 1 and among these APR-246 (PRIMA-1^MET^) has entered clinical trials (Phase III trials; NCT03745716) [83,84,85].

Zanche et al. [95] described the molecular mechanism of CP-31398 and STIMA-1 (a compound structurally related to CP-31398) in reactivation of mutant p53. They found that both molecules have similar chemical activity as a traditional Michael acceptor and that STIMA-1 is more potent than CP-31398 in suppressing growth of mutant p53-expressing tumor cells. A novel compound structurally different from CP-31398 but with similar capacity to restore the p53 wild-type conformation and function to mutant p53, is MIRA-1. MIRA-1 was identified in a cellular screening of a chemical library, and was shown to react covalently with thiol groups in protein [102,104].

Furthermore Madan et al. demonstrated the effects of a curcumin analogue, HO-3867, on p53 activity in cancer cells and tumor xenografts [99]. Mechanistically, HO-3867 alkylates thiol groups in mutant p53 and restores its wild-type conformation, transcriptional activity, and anticancer function in tumor models.

Another small molecule with the ability to reactivate the mutant p53 protein is KSS-9. This compound is a piperlongumine derivative with an aryl-group inserted at the C-7 position and owns highly electrophilic double bonds that react with nucleophiles, such as cysteine sulfhydryl groups, in Michael addition. Although the mechanism whereby KSS-9 bind to mutant p53 is known, the targets Cys residue remain unclear [101].

Bauer et al. [103] found that small 2-sulfonylpyrimidine molecules, named PK11000, stabilize mutant p53 protein by covalent modification of two cysteines, without compromising DNA binding. These compounds were both mild and selective alkylating agents. In particular, the authors demonstrated that PK11007 exerts strong anticancer activity toward p53-compromised cells, involving up-regulation of p53 target genes and a strong increase in cellular ROS levels [103,105,106].

Lambert et al. discovered that APR-246 is not stable under physiological conditions but it is converted into methylene quinuclidone (MQ) featuring a reactive double bond that can participate in Michael addition reactions [86]. Although it is not known which Cys residues are targeted by APR-246, some molecular modelling studies suggest that Cys 124 and Cys 277 are potential target for MQ [84]. In addition, MQ induces inhibition of cellular thiol-dependent redox systems binding, on one hand, the selenocysteine-containing enzyme TrxR1 and Grx [107,108] and, on the other, GSH with depletion of its level [109,110]. Furthermore, MQ induces tumor cells death through caspase-2 and upregulation of pro-apoptotic p53 target genes, such as BAX, PUMA and NOXA [111].

Based on the capacity of APR-246 to restore the wild-type activity by mutant p53 modification, the research of alternative new molecules is becoming a great challenge. Further studies by NMR and X-ray crystallography are required to identify structural movement of Cys in p53 by compounds such as MQ in order to shed light on how the local structural changes are able to recover the wild-type function. This will provide the background for a rational design of more efficient and selective anticancer drugs able to restore mutant p53.

## 5. Concluding Remarks

The presence of several Cys residues that may respond to the redox state of the cell render this protein particularly susceptible to redox changes in the cellular microenvironment. Cysteine residues can undergo oxidative post-translational changes that may also compromise their interaction with zinc ions, altering the conformational structure of the protein and in some cases even its functionality. Remarkably, this redox regulation of p53 fits into a context in which the wild-type tumor suppressor p53 protein possesses an antioxidant defense capacity by upregulating the expression of several genes with free radical scavenger activity. In contrast, mutated p53 GOF isoforms, in addition to losing interaction with DNA and its regulatory elements, acquire the ability to directly interact with transcription factors or transcriptional repressors by regulating a set of different genes with oncogenic and pro-oxidant functionality. The typical overexpression of mutated forms of p53 in cancer may thus favor a more aggressive phenotype not only due to its oncogenic functionality but also to its ability to further modify its protein structure through oxidative post-translational modifications in redox-sensitive cysteine residues. The use of small molecules capable of restoring the wild-type like conformation in mutated forms of p53 may be a valid approach to block this vicious cycle by limiting the formation of an oxidative microenvironment and restoring the tumor suppressor functionality of p53. A better understanding of these mechanisms may finally reveal new opportunities for currently incurable aggressive cancers.

## Figures and Tables

**Table 1:** Summary of p53-reactivating compounds discussed in the text.

Reactivators—p53 Cys-Targeting	Mutations in p53	Mechanism of Action	Ref.
APR-246 (PRIMA-1^MET^) *Quinuclidinone*	R175H; R273H; D259Y/K286E; K286E; S241F; R273C; P223L/V274F	Michael Addition	[84,86,87,88,89,90,91,92,93,94]
CP-31398 *Styrylquinazoline*	V173A; S241F; R249S; R273H	Michael Addition	[95,96,97,98]
HO-3867 *Diarylidenyl piperidone* *curcumin analogue*	Y163H; R175H; H193R; L194F; Y205F; P223L/V274F; C238Y; N239D; S241F; G245S; G245V; M246I; R248Q; R248W; R249S; R273H; C277F; R280K; E285K	Michael Addition	[99,100]
KSS-9 *Piperlongumine derivative*	R175H	Michael Addition	[101]
MIRA-1 *Maleimide*	R175H; P176Y/R248W; R248Q; R248W; R273H; R273H/P309S; R280K; R282W	Michael Addition	[102]
PK11007 *Sulfonylpyrimidine*	Y220; V143A	Nucleophilic aromatic substitution	[103]
STIMA-1 *2*-*vinylquinazolin*-*4*-(*3H*)-*one*	R175H; R273H	Michael Addition	[95]
